# A Broadband Beamformer Suitable for UUV to Detect the Tones Radiated from Marine Vessels

**DOI:** 10.3390/s18092928

**Published:** 2018-09-03

**Authors:** Shilong Ma, Yilin Wang, Nan Zou, Guolong Liang

**Affiliations:** 1 Acoustic Science and Technology Laboratory, Harbin Engineering University, Harbin 150001, China; msl_paper@163.com (S.M.); wangyilin@hrbeu.edu.cn (Y.W.); liangguolong@hrbeu.edu.cn (G.L.); 2 College of Underwater Acoustic Engineering, Harbin Engineering University, Harbin 150001, China; 3 Key Laboratory of Marine Information Acquisition and Security (Harbin Engineering University), Ministry of Industry and Information Technology, Harbin 150001, China

**Keywords:** broadband beamformer, underwater unmanned vehicle (UUV), tone detection

## Abstract

In this paper, the issue that the underwater unmanned vehicle (UUV) with a sonar array achieves the passive detection of vessel targets by detecting the tones radiated from the targets is considered. The multi-beam low-frequency analysis and recording method is widely applied in a manned sonar system. The sonar operator provides an auxiliary decision to extract the target tones from the multiple beams output. However, the complexity of the multi-dimensional information fusion makes it difficult to apply the multi-beam processing in the unmanned sonar system. Aiming at this problem, we introduce the self-adjusting characteristics of adaptive line enhancer to a time domain broadband beamformer and then propose a self-steering broadband beamformer. The proposed beamformer can adaptively steer the main beam to the direction-of-arrival (DOA) of the tonal target. There is no need to pre-form the multiple beams. The complexity of the UUV-based tone detection is reduced. Theoretical derivation and simulation experiments verify that the main beam of the proposed beamformer can track the DOA of tonal target which is rapidly changing. Meanwhile, the tonal interferences as well as the wide-band noise are well suppressed.

## 1. Introduction

With the increasing emphasis on the stealth performance of warships, submarines, underwater vehicles, etc., the radiation noise level of the marine vessels, especially the high-frequency radiation noise level, has been greatly reduced. The target detection utilizing the energy information of the radiation noise has limited performance. However, noise reduction technique is difficult to effectively suppress the low-frequency tonal components of the radiation noise. The low-frequency tones have received extensive attention due to their high signal-to-noise ratio (SNR) per unit bandwidth, small channel propagation loss, and good stability [1,2]. The tones radiated from the marine vessels are of great significance for sonar systems to detect vessel targets [3].

**Figure 1:**
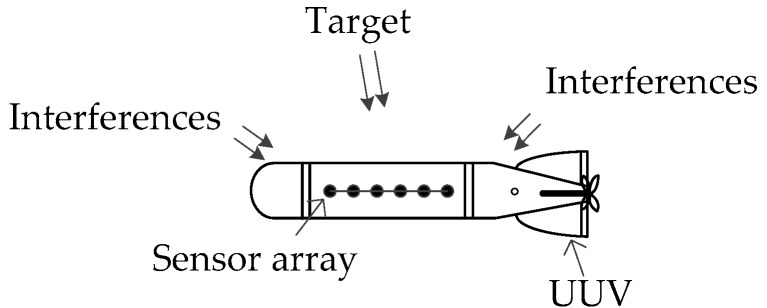
UUV working status.

In recent years, underwater unmanned vehicle (UUV) has attracted much attention [4,5]. The UUV with a sonar array achieves the passive detection of vessel targets by detecting the tones radiated from the targets. The UUV working status is shown in Figure 1. The working background is summarized as follows:

(a) Target signals: The direction, frequency, amplitude, and number of tones are unknown. The total energy of tones is low, but the SNR per unit bandwidth is high.
(b) Interferences: The interferences are composed of the broadband noise and unknown tonal signals radiated from the local platform, which vary with the working status of the platform.
(c) Unmanned mobile platform: Due to mission requirements, the platform or target often performs rotation, fast maneuvering, etc., which causes the direction-of-arrival (DOA) of target to change rapidly.

Under the above complicated conditions, the UUV-based target tone detection faces many challenges.

To improve the ability of the sonar system to detect the tonal signals and suppress the interferences as well as noise, the broadband beamforming techniques, such as conventional frequency domain broadband beamformer (CB) [6], standard Frost time-domain broadband beamformer (SFB) [7], robust Frost beamformer using diagonal loading (DL-RFB) [8,9], time-domain broadband beamformer without pre-steering [10,11], etc., are widely used. However, the above-mentioned broadband beamforming techniques usually require the prior information of target direction which is unknown in our situations. At present, a convenient and effective method is to pre-form the multiple beams covering the entire viewing area, which is widely applied in a manned sonar system. The sonar operator provides an auxiliary decision to extract the target tones from the multiple beams output [12,13,14]. However, the complexity of the multi-dimensional information fusion in time-frequency-direction makes it difficult to apply the multi-beam processing in the unmanned sonar system. Especially when the target DOA changes rapidly, the target signal appears in the multiple beam outputs in turn. It is not conducive for the automatic detection of the target tones.

Considering that the multi-beam processing is not beneficial for the UUV-based tone detection, this paper introduces the self-adjusting characteristics of adaptive line enhancer (ALE) to the time-domain broadband beamformer and then proposes a self-steering broadband beamforming algorithm [15]. The proposed beamformer adaptively steers the main beam to the DOA of the tonal target in the absence of the information such as target direction, frequencies of tones, target motion status, etc. There is no need to pre-form the multiple beams. The complexity of the UUV-based tone detection is reduced. Meanwhile, the tonal interferences and wide-band noise are well suppressed.

## 2. Array Signal Reception Model

**Figure 2:**
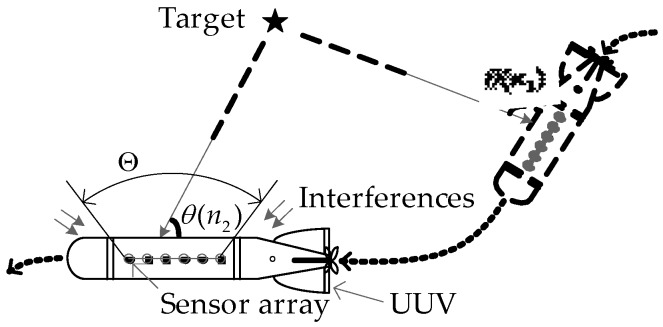
Signal reception diagram for UUV sonar array.

Figure 2 shows a linear sonar array with *M* sensors mounted on a moving UUV. We assume that an unknown tonal target is located in a pre-set subregion of interest Θ. The target signal that impinges on the sensor array is in plane wave form. The received signal at the *m*-th sensor is modelled as follows [16]:

(1) $$x_{m}(n)=s\left[nT_{s}+\tau_{m}\left(\theta (n)\right)\right]+i_{m}(n)+g_{m}(n),\;m=0,1,\ldots ,M-1,$$

where $T_{s}$ denotes the sampling interval. s(t) denotes the tonal signals radiated from the target. $\tau_{m}\left(\theta (n)\right)$ denotes the target signal delay on the *m*-th sensor with respect to the reference sensor, which varies with the unknown DOA of tonal target θ(n). In the case of UUV or target motion (e.g., the fast maneuvering of UUV shown in Figure 2), θ(n) is usually rapidly time-varying. $i_{m}(n)$ denotes the tonal signals located outside Θ (shown as interferences), and $g_{m}(n)$ denotes the broadband Gaussian noise.

## 3. Multi-Beam Lofargram

**Figure 3:**
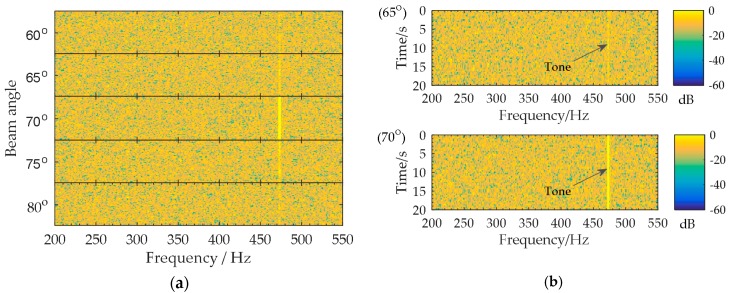
Multi-beam lofargram analysis: (**a**) Multi-beam lofargram; (**b**) The lofargrams in (**a**) corresponding to the beam angles of 65° and 70°.

Low frequency analysis recording (LOFAR) is a classic passive sonar signal processing method in which the tones are detected in a time-frequency spectrogram (i.e., the “lofargram”) [17,18]. The lofargram is obtained by the short-time Fourier transform (STFT) of the received signal. The multiple beams that cover the entire viewing area are formed. The lofargram is calculated at each beam output. Then, a multi-dimensional output with time, frequency, and direction, namely “multi-beam lofargram”, is obtained [12,13,14]. For example, a target tone of 475 Hz appears constantly in the direction of 70° for 20 s. Figure 3a shows the multi-beam lofargram in which the lofargrams of the beam output signals are arranged according to the beam angles. The lofargrams in Figure 3a corresponding to the beam angles of 65° and 70° are shown separately in Figure 3b. It can be seen from Figure 3 that the target tone of 475 Hz appears in the 70° lofargram throughout the entire observation time (0~20 s), while the tone output at other beam angles (for example 65° in Figure 3b) is much weaker. Multi-beam lofargram providing the time-frequency information of the beam output signals in different directions is widely applied in a manned sonar system, where tones are picked out from the multi-beam lofargram using the human eyes.

**Figure 4:**
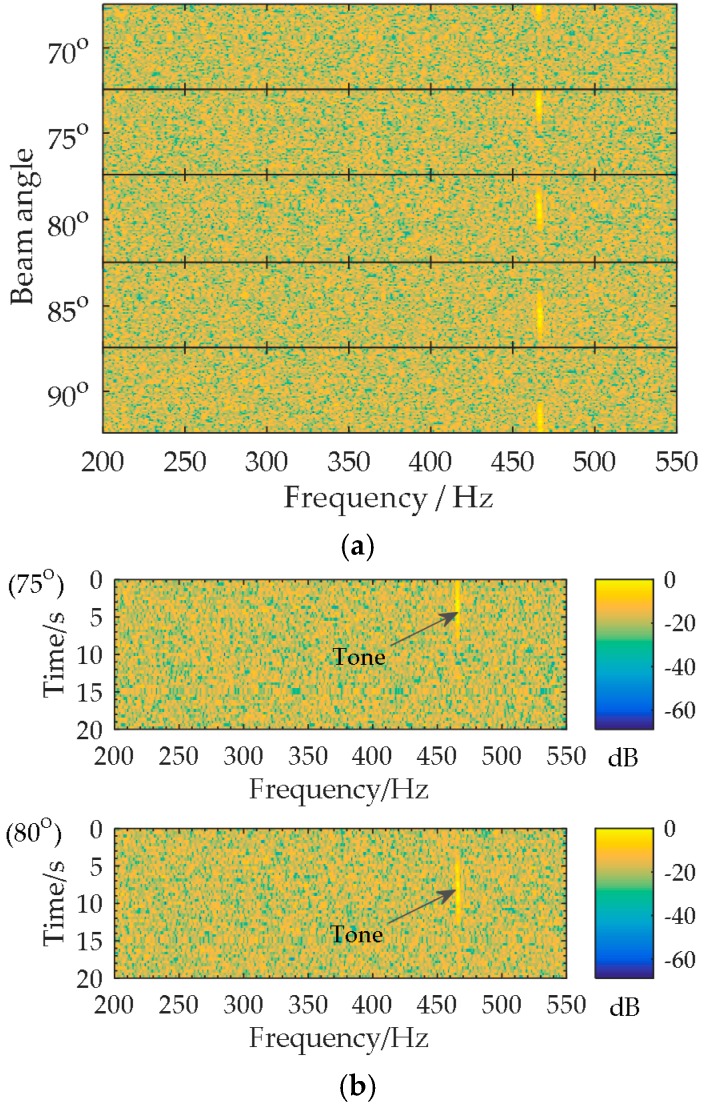
The multi-beam lofargram with a fast variation of target DOA: (**a**) Multi-beam lofargram; (**b**) The lofargrams in (**a**) corresponding to the beam angles of 75° and 80°.

However, the application of the multi-beam lofargram on UUV has the following problems: Firstly, the complexity of the multi-dimensional information fusion in time-frequency-direction is not conducive for the automatic detection of target tones. Secondly, the DOA of the tonal target is usually rapidly time-varying due to the fast maneuvering of UUV or target, which causes the target tone to appear intermittently in the lofargrams in different directions. In Figure 4, as the DOA of the target rapidly varies from 70° to 90° in 0~20 s, the tone of 475 Hz appears in the 75° lofargram at the observation time 0~7 s, and then the tone presents in the 80° lofargram during 7~14 s. The discontinuous appearance of the tone in the multi-beam lofargram further aggravates the difficulty of automatic tone detection. In view of the above difficulties, a special broadband beamforming technique that is suitable for UUV to detect the tones radiated from marine vessels is needed.

## 4. Self-Steering Beamforming Technique for Tone Detection

To solve the above problems, a self-steering broadband beamformer (SBB) for tone detection is proposed in this paper. Firstly, the self-adjusting filter characteristics of ALE are introduced to the time-domain broadband beamformer. Then the beam self-steering is achieved. Secondly, a space-time projection matrix is used to suppress the tonal interferences. The convergence rate of the self-steering beamforming is improved by implementing the beamforming on decimated sub-band signals. The proposed beamformer adaptively steers the main beam to the DOA of the tonal target. The target tones can be extracted from the self-steering beam output. There is no need to pre-form the multiple beams. The complexity of the UUV-based tone detection is reduced. Besides, the tonal interferences and wide-band noise are well suppressed.

### 4.1. Beam Self-Steering

**Figure 5:**
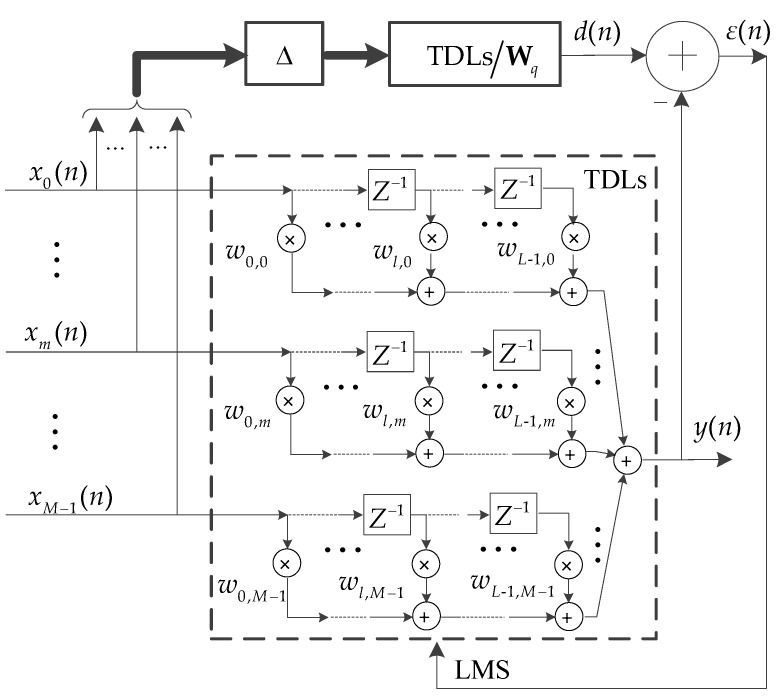
Schematic diagram of beam self-steering.

The beam self-steering is implemented by introducing the self-adjusting filter characteristics of ALE to the time-domain broadband beamformer, as shown in Figure 5. Firstly, a non-external reference signal d(n) of the target tones is obtained from the received signal utilising the coherence property of tone. Secondly, the beam self-steering property is realised by minimising a mean squared error (MSE) between d(n) and the beam output y(n). The least-mean-square (LMS) algorithm is used to solve the minimum MSE and update the weight vector of the beamformer.

#### 4.1.1. Time-Domain Broadband Beamformer Output y(n)

The Frost time-domain broadband beamformer consists of two parts: The pre-steering delays and the multi-channel tapped delay lines (TDLs) [10]. Since TDL has the capability of time delay [19], the pre-steering delays are integrated into the TDLs. Then, a time-domain broadband beamformer structure without pre-steering delays (i.e., the TDL structure in Figure 5) is obtained [10]. The beamformer structure without pre-steering delays is employed in this paper, which is expressed as

(2) $$y\left(n\right)=W^{H}(n)X\left(n\right),$$

(3) $$X(n)=[x_{0}(n),\cdots ,x_{M-1}(n),x_{0}(n-1),\cdots ,x_{M-1}(n-1),\cdots ,{x_{M-1}(n-L+1)]}^{T},$$

(4) $$W(n)=[w_{0,0}(n),\cdots ,w_{0,M-1}(n),w_{1,0}(n),\cdots ,w_{1,M-1}(n),{\cdots ,w_{L-1,M-1}(n)]}^{T}.$$

The main beam is steered towards the target DOA by adaptively updating the weight vector W(n). The target tones can be extracted from y(n).

#### 4.1.2. Acquisition of the Reference Signal d(n)

**Figure 6:**
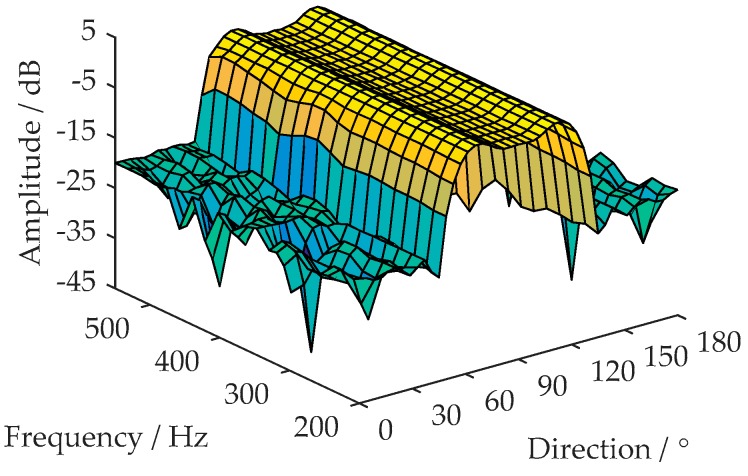
Magnitude response of the fixed weight vector $W_{q}$.

According to the principle of ALE [15], the characteristic that the tone has a larger coherence radius than the broadband noise is utilized. The reference signal d(n) is obtained as

(5) $$d(n)=W_{q}^{T}X(n-\Delta ),$$

(6) $$W_{q}={\left[w_{q\_0,0},\cdots ,w_{q\_0,M-1},w_{q\_1,0},\cdots ,w_{q\_1,M-1},\cdots ,w_{q\_L-1,M-1}\right]}^{T},$$

d(n) is the interference-free and Δ-delayed version of the received signal X(n). Sufficient time delay Δ decorrelates the broadband components between X(n) and d(n), while keeping the tonal components correlated [15]. Here, a TDL structure is employed to eliminate the tonal interferences outside Θ. $W_{q}$ is a fixed weight vector of the TDLs structure. Figure 6 shows an example of the $W_{q}$ amplitude response. The design of $W_{q}$ is formulized into a convex optimization problem as shown in Equation (7). The optimization problem is solved by utilizing the convex optimization tool [20].

(7) $$s.t.\begin{array}{l} \underset{W_{q}}{\min}\;\underset{f,\theta }{\max}\left|W_{q}^{T}a\left(f,\theta \right)-1\right|,\;\forall f\in \Omega ,\forall \theta \in \Theta \\ \left|W_{q}^{T}\left[a\left(f,\theta \right)-a\left(f_{0},\theta \right)\right]\right|\leq \delta_{\Theta },\;\forall f\in \Omega ,\forall \theta \in \Theta \\ \left|W_{q}^{T}a\left(f,\theta \right)\right|\leq \delta_{\bar{\Theta }},\;\forall f\in \Omega ,\forall \theta \in \bar{\Theta }\\ \left\|W_{q}^{T}\right\|\;\leq \delta_{\mathrm{norm}} \end{array},$$

where $\delta_{\Theta }$ is the constraint factor of constant beamwidth and $f_{0}$ is the reference frequency. $\bar{\Theta }$ denotes the stopband subregion. $\delta_{\bar{\Theta }}$ is the constraint factor of stopband attenuation degree. $\delta_{\mathrm{norm}}$ is a small positive value. Ω denotes the system bandwidth. a(f,θ) is LM×1 steering vector and is given by

(8) $$a\left(f,\theta \right)=\left[{\tilde{a}}_{0}^{T}\left(f,\theta \right),\ldots ,{\tilde{a}}_{m}^{T}\left(f,\theta \right),\ldots ,{\tilde{a}}_{M-1}^{T}\left(f,\theta \right)\right],$$

(9) $${\tilde{a}}_{m}\left(f,\theta \right)={\left[a_{0,m},\ldots ,a_{l,m},\ldots ,a_{L-1,m}\right]}^{T},$$

(10) $$a_{l,m}=\cos\left[2\pi f\left(lT_{s}+\tau_{m}\left(\theta \right)\right)\right],\;l=0,1,\ldots ,L-1,m=0,1,\ldots ,M-1.$$

#### 4.1.3. Adaptive Adjustment of the Weight Vector W(n)

The target tones in y(n) have the optimal output SNR, when the MSE between y(n) and the reference signal d(n) reaches a minimum value. At this point, the main beam is steered towards the DOA of the tonal target θ(n), which is a necessary condition for achieving the optimal output SNR of target tones. Hence, the adaptive weight vector W(n) that steers the main beam towards θ(n) is obtained from the minimisation of the MSE, as shown in Equation (11).

(11) $$W(n)=\arg\underset{W(n)}{\min\;}E\left\{{\left|d(n)-W^{H}\left(n\right)X\left(n\right)\right|}^{2}\right\}$$

The LMS algorithm is utilised to solve Equation (11) [15]. W(n) is updated to converge on the optimal solution of (11) by

(12) W(n+1)=W(n)+με(n)X(n),

where $\varepsilon (n)=d(n)-W^{H}\left(n\right)X\left(n\right)$ is the residual error, and μ is the step size. To ensure the convergence of the algorithm, the step size μ is bounded by [15]

(13) $$0<\mu <1/\lambda_{\max\;}$$

where $\lambda_{\max}$ denotes the largest eigenvalue of the covariance matrix $R=E\left[X(n)X^{H}(n)\right]$. A smaller step size μ can reduce the steady-state error, but the convergence rate of the algorithm will decrease. The choice of the step size μ requires a trade-off between convergence rate and steady-state error.

#### 4.1.4. Beam Self-Steering Characteristics Analysis

In Equation (1), let

(14) $$s\left[nT_{s}+\tau_{m}\left(\theta (n)\right)\right]=\sum_{k=1}^{K}A_{k}\cos\left[2\pi f_{k}(n)\left(nT_{s}+\tau_{m}\left(\theta (n)\right)\right)\right],$$

where *K* is the number of target tones. $A_{k}$ and $f_{k}\left(n\right)$ denote the amplitude and frequency of a target tone, respectively. $\tau_{m}\left(\theta (n)\right)=d\sin\left(\theta (n)\right)m/C$, *d* denotes the space of adjacent sensors, and *C* denotes the underwater sound velocity. For the convenience of analysis, we assume that $f_{k}\left(n\right)$ and θ(n) are constant during *LM* sampling periods. The $g_{m}(n)$ in Equation (1) is an isotropic Gaussian noise with variance $\sigma_{0}^{2}$. Utilising the undetermined coefficient method described in Reference [21], the optimal solution $W^{*}(n)$ of Equation (11) is analytically expressed as

(15) $$\begin{array}{c} w_{l,m}^{*}\left(n\right)=\sum_{k=1}^{K}\frac{2\sigma_{k}^{2}}{2\sigma_{0}^{2}+LM\sigma_{k}^{2}}\cos[2\pi f_{k}(n)\left(t_{d}-\tau_{m}\left(\theta (n)\right)+l/f_{s}-\Delta /f_{s}\right)],\\ \left(l=0,1,\ldots ,L-1,\;m=0,1,\ldots ,M-1\right) \end{array}$$

where $\sigma_{k}^{2}$ is the variance of the tone, and $f_{s}=1/T_{s}$ is the sampling rate. $t_{d}$ is a fixed delay value. The beam response function of $W^{*}(n)$ is given by

(16) $$\begin{array}{l} B_{n}\left(\varphi ,\omega \right)=\sum_{l=0}^{L-1}\sum_{m=0}^{M-1}w_{l,m}^{*}(n)e^{-j\omega l}e^{-j\varphi m}\\ =\sum_{k=1}^{K}\frac{\sigma_{k}^{2}}{\left(2\sigma_{0}^{2}+LM\sigma_{k}^{2}\right)}\frac{\sin\left[\left(\varphi_{k}(n)-\varphi \right)M/2\right]}{\sin\left[\left(\varphi_{k}(n)-\varphi \right)/2\right]}\frac{\sin\left[\left(\omega_{k}(n)-\omega \right)L/2\right]}{\sin\left[\left(\omega_{k}(n)-\omega \right)/2\right]},\\ \times e^{j\left(\varphi_{k}(n)-\varphi \right)\left(M-1\right)/2}e^{j\left(\omega_{k}(n)-\omega \right)\left(L-1\right)/2}e^{-j2\pi \omega_{k}(n)\left(\Delta -t_{d}f_{s}\right)} \end{array}$$

where φ=2πfsin(θ)d/C denotes the spatial frequency, and $\omega =2\pi f/f_{s}$ denotes the angular frequency. $\varphi_{k}(n)=2\pi f_{k}(n)d\sin\left(\theta (n)\right)/C$ is the tonal phase difference between the adjacent sensors. $\omega_{k}(n)=2\pi f_{k}(n)/f_{s}$ denotes the digital frequency of the tonal signal.

The real-time update in Equation (12) converges W(n) on the optimal solution $W^{*}(n)$. Thus, the beam response function of W(n) approximates Equation (16). Equation (16) serves as the response function of a space-time filter in which its passband matches with the DOA θ(n) ($\varphi_{k}(n)$) and frequency $f_{k}$ ($\omega_{k}$) of each unknown target tone. This is, the self-steering beam is adaptively steered to the unknown target tones. The broadband noises and tonal interferences that fall in the filter stopband are attenuated. With the increase of *M* and *L*, the filter bandwidth becomes narrower, and the ability to suppress the interferences and broadband noises is stronger. But, the weight vector noise becomes larger.

### 4.2. Interference Suppression and Convergence Rate Improvement

After the process described in Section 4.1, the beam self-steering characteristics are obtained. However, there are two problems in the practical application of the self-steering beamforming: (a) With the increase of *M* and *L*, it is difficult for the self-steering beamforming to reconcile the contradiction between maladjustment noise and convergence rate. In addition, the convergence rate of the self-steering beamforming decreases in a color noise background. (b) The side-lobe attenuation of the self-steering beam response has a limited ability to suppress the strong tonal interferences. Aiming at the above problems, the convergence rate of the self-steering beamforming is improved by performing the beamforming on sub-band signals. A space-time projection matrix is used to suppress the strong tonal interferences.

A. Sub-band beamforming

The spectral flatness of the sub-bands usually exceeds that of the full-band [22]. The tap number *L* and the update rate of the weight vector decrease for a low rate sampling signal. Therefore, the self-steering beamforming is performed on decimated sub-band signals, namely the sub-band adaptive filter (SAF) processing [23,24,25]. Then the maladjustment noise is reduced, and the convergence rate of the self-steering beamforming is improved. In addition, normalized least mean square (NLMS) is used instead of the LMS algorithm to further improve the convergence rate of the self-steering beamforming.

Firstly, the sub-band signals are decomposed from the received signal and decimated by the sub-band number. Secondly, the self-steering beamforming in Figure 5 is employed in each sub-band signal. Meanwhile, the NLMS is used instead of the LMS algorithm. Finally, all the sub-band beam outputs are fused into one beam output by up-sampling and synthesis filtering. Cosine modulated filter bank (CMFB) which can efficiently implement the sub-band filtering processing is used for the sub-band decomposition and signal synthesis in this paper [26]. The CMFB consists of analysis filter bank and synthesis filter bank. The analysis filter bank implements the sub-band filtering and decimation of the received signal. The synthesis filter bank performs the up-sampling and synthesis filtering on the sub-band beam outputs [26].

B. Space-time projection matrix

We define an LM×LM matrix

(17) $$G=\iint_{\Omega \;\Theta \;}a\left(f,\theta \right)a^{T}\left(f,\theta \right)dfd\theta ,$$

where a(f,θ) is the LM×1 steering vector described in Equation (8). Θ  is the subregion of interest. Ω is the system bandwidth. ${\left\{u_{p}\right\}}_{p=1}^{P}$ denotes the *P* main eigenvectors of $G\in {\mathbb{C}}^{LM\times LM}$. $B=\left[u_{1},u_{2},\ldots ,u_{P}\right]$ spans a signal subspace that approximates the subspace extended by all the a(f,θ) in Θ  and Ω [27]. The space-time projection matrix is calculated as $BB^{H}$. The W(n) is multiplied by $BB^{H}$ to suppress the interferences outside Θ  and Ω. Thus, the algorithm has a general two-stage structure [28,29].

**Figure 7:**
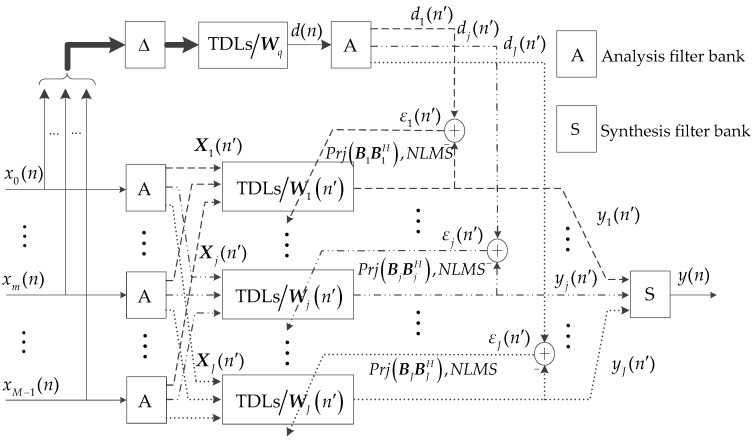
Schematic diagram of the proposed SBB.

With the sub-band decomposition and the space-time projection matrix $BB^{H}$, the schematic diagram of the proposed SBB is shown in Figure 7. The processing flow is as following: Firstly, using the analysis filter bank, $x_{m}(n)$ and d(n) are respectively decomposed into *J* sub-band signals and decimated by *J*. $X_{j}(n^{\prime })$ denotes the *j*-th sub-band received signal. $d_{j}(n^{\prime })$ denotes the *j*-th sub-band reference signal. $1,2,\ldots ,n^{\prime }$ is the decimated time series. Secondly, the self-steering beamforming in Figure 5 is employed in each sub-band signal $X_{j}(n^{\prime })$. The *j*-th sub-band beam output is expressed as $y_{j}(n^{\prime })=W_{j}^{H}\left(n^{\prime }\right)X_{j}(n^{\prime })$. The iteration formula for the weight vector $W_{j}\left(n^{\prime }\right)$ is

(18) $$W_{j}\left(n^{\prime }+1\right)=W_{j}\left(n^{\prime }\right)+\mu \varepsilon_{j}(n^{\prime })B_{j}B_{j}^{H}X_{j}(n^{\prime })/\left(X_{j}^{H}(n^{\prime })X_{j}(n^{\prime })\right),$$

where $\varepsilon_{j}(n^{\prime })=d_{j}(n^{\prime })-W_{j}^{T}\left(n^{\prime }\right)X_{j}(n^{\prime })$ is the residual. $X_{j}^{H}(n^{\prime })X_{j}(n^{\prime })$ is the energy normalization term for the NLMS algorithm. $B_{j}B_{j}^{H}$ denotes the *j*-th sub-band space-time projection matrix. Finally, the sub-band beam outputs $y_{j}(n^{\prime })$ are fused into the beam output y(n) by the synthesis filter bank.

The construction process of $B_{j}B_{j}^{H}$ is as follows: The a(f,θ), f∈Ω, θ∈Θ  in Equation (8) is grouped according to the sub-band $\psi_{j}=\left(jf_{s}/\left(2J\right),\;\left(j+1\right)f_{s}/\left(2J\right)\right]\subseteq \Omega$, *j* = 0, 1, …, *J* − 1. The vector ${\tilde{a}}_{m}\left(f,\theta \right)$ in a(f,θ) is decimated by *J*. Then the decimated sub-band steering vector $a_{j}\left(f,\theta \right)$, $f\in \psi_{j}\subseteq \Omega$ is obtained. The matrix $G_{j}$ for each sub-band is given by

(19) $$G_{j}=\iint_{\psi_{j}\;\Theta \;}a_{j}\left(f,\theta \right)a_{j}^{H}\left(f,\theta \right)dfd\theta .$$

$B_{j}$ is composed of the main eigenvectors of $G_{j}$. The sub-band space-time projection matrix is calculated as $B_{j}B_{j}^{H}$.

In conclusion, the implementation steps of the proposed SSB algorithm are summarized as follows:

1. Initial design: $W_{q}$ is designed by Equation (7). The sub-band space-time projection matrix $B_{j}B_{j}^{H}$ is calculated from Equation (19).
2. Calculate the target reference signal d(n) according to Equation (5).
3. $x_{m}(n)$ and d(n) are respectively filtered by the analysis filter bank. The decimated sub-band signals $X_{j}(n^{\prime })$ and $d_{j}(n^{\prime })$ are obtained.
4. Calculate the sub-band beam output $y_{j}(n^{\prime })=W_{j}^{H}\left(n^{\prime }\right)X_{j}(n^{\prime })$ and update the $W_{j}\left(n^{\prime }\right)$ by Equation (18).
5. The sub-band beam outputs $y_{j}(n^{\prime })$ are fused into the beam output y(n) by the synthesis filter bank. Repeat the steps 2, 3, 4, 5, until all received signals are processed.

## 5. Simulations

### 5.1. Broadband Beam Response Analysis

When the DOA of tonal target rapidly changes, the broadband beam response of the SBB is analyzed to evaluate the beam self-steering performance. Assume a uniform linear array with *M* = 20 sensors with a space of *d* = 1.36 m. The underwater sound velocity *C* = 1500 m/s. Sampling rate $f_{s}$ is 2.5 kHz. The subregion of interest Θ  is set to 50~130°. A tonal target with three tones of 495 Hz, 475 Hz, and 415 Hz is located inside Θ , and the SNR of each tone is −20 dB. The target DOA varies from 70° to 120° over 17 s. In addition, a tonal interference is located in 160°, whose tonal frequencies are 360 Hz and 440 Hz. The interference-to-noise ratio of each tone is 5 dB. The system bandwidth Ω is 200 Hz~550 Hz. The sub-band number of the SSB is *J* = 64. The number of TDL nodes is *L* = 30. The step size of the NLMS algorithm in each sub-band is 0.1. Delay Δ is set to 0.01 s.

**Figure 8:**
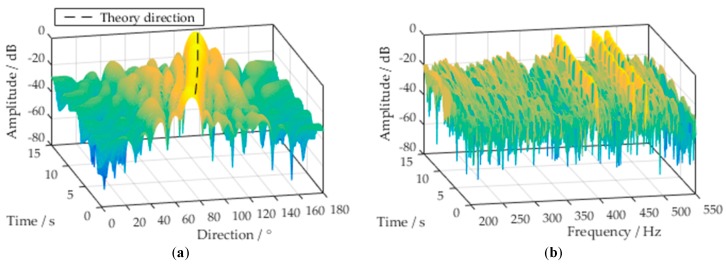
Beam response of the SBB: (**a**) Time-space response at tonal frequency; (**b**) Time-frequency response at target direction.

Figure 8a shows the beam response in time-space domain at the tonal frequency of 415 Hz. The main beam direction of the SBB coincides with the theoretical direction of the target. This implies that the main beam of the SBB autonomously points to the target DOA and changes with it. Due to the role of the space-time projection matrix, the beam response of the SBB outside Θ  has a large attenuation to suppress the interferences. Figure 8b shows the beam response in time-frequency domain at the target direction. There is a large amplitude response at the target tonal frequency (e.g., 415 Hz). The amplitude response at the tonal interference (e.g., 360 Hz) is small. The broadband beam automatically matches the target tones in frequency domain. Then, the broadband background noise and tonal interferences are suppressed.

### 5.2. Lofargram Analysis of the Beam Output

Under the simulation conditions in Section 5.1, the lofargram of the SBB beam output is calculated in this section. The convenience and efficiency of the SBB for tone detection are illustrated by comparison with the multi-beam lofargram. The data length of the STFT in the lofargram is 1000.

**Figure 9:**
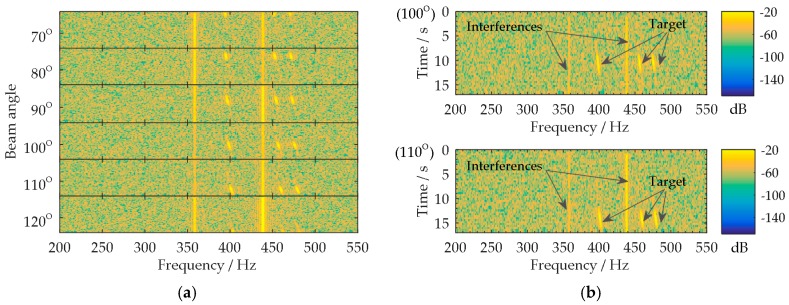
Multi-beam lofargram analysis: (**a**) Multi-beam lofargram; (**b**) The lofargrams in (**a**) corresponding to the beam angles of 100° and 110°.

**Figure 10:**
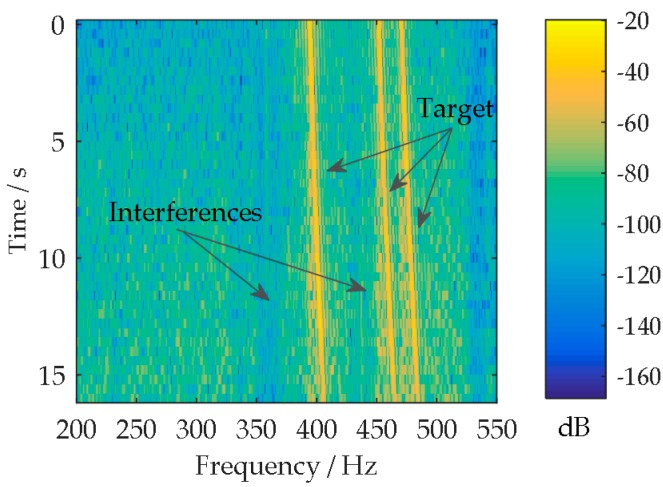
The lofargram of the SBB beam output.

The multi-beam lofargram is shown in Figure 9a. The lofargrams in Figure 9a corresponding to the beam angles of 100° and 110° are shown separately in Figure 9b. As the target DOA changes rapidly, the three target tones (nearby 495 Hz, 475 Hz, and 415 Hz) appear in the 100° lofargram at the observation time 8~12 s, and then the tones present in the 110° lofargram during 12~16 s. The target tones appear in each beam angle for a short time. The appearance duration is related to the beam width and the variation rate of target DOA. It is not convenient for the autonomous detection of the target tones. Figure 10 shows the lofargram of the SBB beam output. Since the main beam of the SBB tracks the changes of target DOA, the three target tones (nearby 495 Hz, 475 Hz, and 415 Hz) continuously appear throughout the observation time 0~17 s in the lofargram. The target tones can be easily extracted from the self-steering beam output. Thus, the complexity of the UUV-based tone detection is reduced. Meanwhile, the SBB beam output has lower broadband noise background and weaker tonal interferences (nearby 360 Hz and 440 Hz).

### 5.3. The Beam Output Performance for Different DOAs of Target

The curve of the output signal-to-interference-plus-noise ratio (SINR) versus the DOA of the target is analyzed to evaluate the beam output performance of the SBB. Meanwhile, the SBB is compared with the beamforming methods of CB, SFB, and DL-RFB. The tonal interference lies in 160°, and the interference-to-noise ratio of each tone is 20 dB. The DOA of target is in the range of 70~110°, and the SNR of each tone is −15 dB. The main beam direction of CB, SFB, and DL-RFB is steered to 90° (the abeam direction). The output SINR is calculated by 100 Monte Carlo trials. The diagonal loading value of the DL-RFB is five times the variance of the input noise. Other conditions are the same as in Section 5.1.

**Figure 11:**
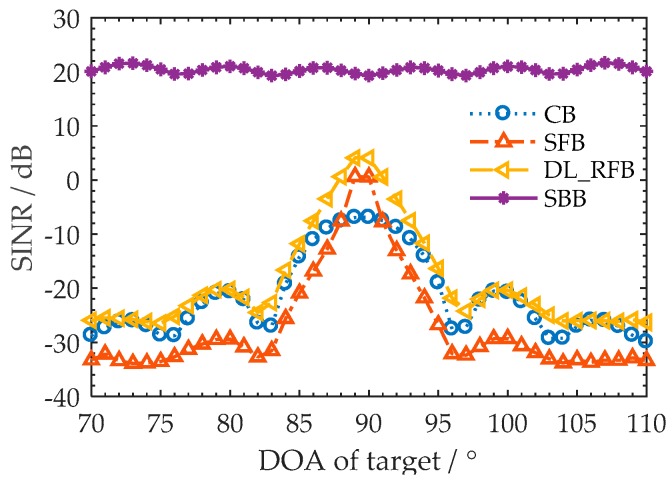
Output SINR versus DOA of target.

The curves of the output SINR versus the DOA of target are shown in Figure 11. The deviation between the main beam direction and the DOA of the target is called as the “look direction deviation”. As the DOA of the target changes from 90° to 95° (or to 85°), the look direction deviation of CB, DL-RFB, and SFB increases to 5°, which leads to a rapid decline in the output SINRs. However, the output SINR of the SBB remains relatively constant (stabilized at around 20 dB) for the different DOAs of the target (70~110°). The reason for this is that the SBB adaptively steers the main beam to the DOA of the tonal target. The look direction deviation is avoided, which results in its good robustness of output SINR. In addition, the SBB effectively suppresses the broadband noise as well as the tonal interferences. Thus, a higher output SINR than other methods is obtained.

## 6. Conclusions

In this paper, we propose a self-steering broadband beamformer for tone detection, which is suitable for UUV to detect the tones radiated from marine vessels. The self-adjusting filter characteristics of ALE are introduced to the time-domain broadband beamformer. Then the beam self-steering is achieved. A space-time projection matrix is used to suppress the tonal interferences. The convergence rate of the self-steering beamforming is improved by implementing the beamforming on decimated sub-band signals. Theoretical derivation and simulation experiments verify that the proposed beamformer adaptively steers the main beam to the DOA of the tonal target in the absence of the information such as target direction, frequency of tone, target motion status, etc. The target tones can be extracted from the self-steering beam output. There is no need to pre-form the multiple beams. The complexity of the UUV-based tone detection is reduced. In addition, the tonal interferences and the wide-band noise are well suppressed.
